# The role of cooperatives in sustaining the livelihoods of rural communities: The case of rural cooperatives in Shurugwi District, Zimbabwe

**DOI:** 10.4102/jamba.v9i1.341

**Published:** 2017-04-24

**Authors:** Smart Mhembwe, Ernest Dube

**Affiliations:** 1 Department of Development Studies, Zimbabwe Open University, Zimbabwe; 2 Department of Development Studies, Midlands State University, Zimbabwe

## Abstract

The main focus of the research was to analyse the role of cooperatives in sustaining the livelihoods of local rural communities in Shurugwi District in Zimbabwe. Descriptive survey design was used in this mixed method approach to the study. A questionnaire, interviews and observation methods were employed as the main research instruments. Purposive sampling technique was adopted and data were collected from government officials and from members of the six cooperatives in Shurugwi District. A total of 50 research participants were involved in the study. It was found that cooperatives were established as a strategy to sustain livelihoods of rural communities. With the adoption of cooperatives, people in the rural communities managed to generate employment, boost food production, empower the marginalised, especially women, and promote social cohesion and integration, thereby improving their livelihoods and reducing poverty. Most cooperatives face a number of challenges that include lack of financial support, poor management and lack of management skills, and lack of competitive markets to sell their produce. The study recommends that the government and the banking sector render financial support to cooperatives in rural communities to allow them to expand and diversify their business operations; constant training on leadership and management skills is provided to cooperatives’ members. There is also a need for cooperatives, especially those in the agricultural sector, to form some producer associations so as to easily market their produce. Lastly, the study recommends that future research should focus on investigating issues that hinder the growth of the cooperative movement in rural communities of Zimbabwe. It is hoped that policy-makers, the academia and communities would benefit from the study.

## Introduction

Cooperatives play a significant role in improving the livelihoods of rural communities the world over. Ortmann and King (2007) observe that cooperatives originated in Europe, before they spread to other industrialised countries during the late 19th century. However, the development of these cooperatives was taken as a measure to counter extreme conditions of poverty. In the African continent, Kenya is one of the countries with the longest history of cooperative development that has been characterised by strong growth, such that it has made significant contributions to the overall economy of the nation since it attained its independence. According to Wanyama (2009), in 2009, the Ministry of Development and Marketing in Kenya established that 80% of Kenya’s population was deriving its income either directly or indirectly from activities of cooperatives.

Schwettmann (2004) notes that at least 40% of the households in Africa are members of cooperative societies. Thus, when taken as a whole, the cooperative movement is taken to be Africa’s biggest non-governmental organisation (NGO). The cooperatives play a significant role in many national economies and have created a great number of self-employment opportunities in Africa. An International Labour Organisation (ILO) study in 1997 estimated that the cooperative sector in 15 African countries was responsible for nearly 160 000 direct jobs (Wanyama, Develtere & Pollet 2008). This shows that cooperatives help in the creation of employment opportunities. There are three different ways in which cooperatives can create employment. Firstly, cooperatives offer direct wage employment to people who work in both primary and secondary cooperatives. Secondly, cooperatives offer self-employment to their members, whose participation in the economic activities makes it possible for them to be guaranteed a decent income. Thirdly, cooperatives indirectly employ other people through the spillover effect of cooperatives’ activities to non-members, who generate income through transactions and opportunities created by cooperatives. However, Schwettmann (2004) further observes that the role of cooperatives in creating employment has been formerly neglected by employment planners, cooperative promotion agencies, social partners and the donor organisation alike.

However, looking at the Zimbabwean context, one can note that the country has been experiencing an economic downturn over the last two decades as a result of recurring droughts resulting from climate change, political instability and the devastating impact of the HIV and/or AIDS pandemic. As a result of such prevailing conditions in Zimbabwe, rural cooperatives are becoming a force to reckon with as they have a potential to boost food security, increase employment opportunities and improve households incomes. As such, this study sought to identify the role of cooperatives in sustaining the livelihoods of rural communities in Shurugwi District in Zimbabwe. The objectives of the study were, therefore, to identify the reasons for the establishment of rural cooperatives in the district, to assess the role of rural cooperatives in improving the livelihoods of rural communities and to analyse the challenges faced by rural cooperatives in their attempt to address the socio-economic challenges faced by rural households.

## Statement of the problem

There has been a problem of viable economic sectors in most rural communities in Zimbabwe and Shurugwi District in the Midlands Province is no exception. The district’s food security situation has been under threat owing to recurrent drought resulting from climate change, political polarisation or instability and the devastating effects of the HIV and/or AIDS pandemic. NGOs have tried hard to intervene through the promotion of small projects in an attempt to create employment and generate income for rural households. However, these interventions have been found to be unsustainable because of funding challenges faced by these NGOs. The Government of Zimbabwe (GoZ), through the Ministry of Small and Medium Enterprises and Cooperatives Development, has established cooperatives in the district. The Ministry has spearheaded the development of these cooperatives. The rationale behind their establishment was to sustain the livelihoods of rural communities through the creation of employment, resulting in improved income and eradication of poverty from the communities. However, it seems the livelihoods of rural communities in Shurugwi District are still not sustainable despite the creation and the presence of activities of the cooperatives. If this scenario is not addressed, unemployment, food insecurity and poverty levels would increase with greater margins in the district. There is a need for improvement in the role of the cooperatives if the livelihoods of rural communities in the district are to be sustained.

## Literature review

### The concept of the cooperative movement

The International Cooperative Alliance (ICA) (2005:n.p.) defines a cooperative as ‘an autonomous association of persons united voluntarily to meet their common social, economic and cultural needs as well as their aspirations through a jointly owned and democratically controlled enterprise’. In light of the above values, cooperatives can then be described as a distinct, mutual-based association or group with varied capital and membership base which is democratically managed. A cooperative is distinct from a socio-professional body and its mission is to defend the interests of its members or a community development association whose activities are similar to those of a pressure group. Chitsike (1988) notes that a necessary feature of a cooperative is the mutual commitment of each member involved. Every member is responsible for the preservation of its autonomous identity, as an association of people formally engaged in private enterprise with a strict beneficial economic purpose.

#### Principles governing cooperatives

The life and work of a cooperative business are governed by several values and seven major principles that enable cooperatives to be viable (Kumar, Wankhede & Gena 2015; Tchami 2007). The following are the principles that govern cooperatives in putting their values into practice. These are the principle of voluntary and open membership; principle of democratic member control; principle of member economic participation; principle of autonomy and independence; principle of education, training and information; principle of cooperation and, principle of concern for community. The *principle of voluntary and open membership* indicates that cooperatives are open to all persons that are able to use their service and willing to accept the responsibilities of membership without gender, social, racial, political or religious discrimination. Tchami (2007) asserts to this view and states that cooperatives have to accept only a predetermined number of members, depending on the capacity of those cooperatives. The *democratic member control principle* indicates that cooperative societies are democratic organisations, controlled by their members who actively participate in the formulation of policies and in making decisions. As such, cooperatives should be run by men and women, who serve as elected representatives and should be accountable to the general membership. Laidlaw (1980) asserts that in primary cooperatives, the principle of democratic control is noticed when members of the cooperative exercise equal voting rights by virtue of the ‘one member one vote’ rule. What this shows is that cooperatives should be democratically managed.

As far as the *principle of member economic participation* is concerned, members in a cooperative society contribute equitably to the society and democratically control the capital of their cooperative societies (Galor 1995). The *principle of autonomy and independence* views cooperatives as autonomous, self-help organisations that are controlled by their members. Thus, in the event that cooperatives are to enter into agreements with other organisations, for instance, to raise capital from external sources, they should do so on terms that ensure democratic control by their members so as to maintain the autonomy of the cooperative. The *principle on education, training and information*, as noted by Tchami (2007), is the fifth principle and clearly highlights that cooperatives should provide education and training to their members. That is, elected representatives, managers and their employees need to be educated and trained often in order for them to contribute effectively to the development of the cooperative. Ortmann and King (2007:40) add that members of the cooperative should learn how to work together so as to relinquish their personal interests in favour of the interests of the group, whilst the management have to learn how to use their powers wisely in running a democratic commercial enterprise.

Cooperation among members plays a significant role in the success of cooperatives. Thus, the *principle of cooperation* among cooperatives is emphasised. The ICA (2005) notes that cooperatives serve their members most effectively, whilst at the same time strengthening the cooperative movement to work together through local, national, regional and international structures. Therefore, teamwork among members of cooperatives should be the order of the day. The *principle of concern for community* is one major principle guiding the operations and activities of cooperatives. This principle emphasises that cooperatives should work for the sustainable development of their communities through enabling policies which are approved by their members. If properly followed and implemented, these principles would guide cooperatives towards the achievement of their objectives in order to sustain livelihoods of rural communities. The following section highlights the purpose of cooperatives and how they can sustain the livelihoods of rural communities.

#### How cooperatives can sustain livelihoods of communities

Cooperatives exist for different purposes in human societies. As such, they can sustain livelihoods of rural communities in many different ways. Tchami (2007) observes that the main purpose of a cooperative is to allow individuals to come together and pool their resources in order to reach a common goal, which would be difficult for them to achieve as individuals. Furthermore, cooperation in most cases occurs when external factors threaten a certain number of individuals; hence, cooperatives are the best possible means of defence against the worsening social and economic conditions affecting a section of the population. In this way, cooperatives are enterprises which help their members to cooperate in solving social problems they share.

The National Cooperative Business Association (NCBA) (2005) argues that cooperatives are formed by their members, especially when the market fails to provide the much needed goods and services at affordable prices and of acceptable quality. In light of these sentiments, one can note that cooperatives empower people to improve their quality of life and to enhance their economic opportunities mainly through self-help projects. Barton (2000) also states that the major reason why cooperatives are formed is to strengthen the bargaining powers of their members, help them in maintaining access to comparative markets and to capitalise on new market opportunities. As such, they can easily obtain the much needed products and services on competitive basis, whilst at the same time they improve income opportunities, reduce costs and manage risks of the members.

Attwood and Baviskor (1980) argue that most of the rural cooperatives, especially those that were established as a result of government policies in the developing world, sought to bridge the gap that existed between rural and urban areas. What this reflects is that rural cooperatives sought to rectify the colonial legacy of economic dualism, which was characterised by policies that used to favour the development of urban industrial sectors at the expense of rural areas. Chitsike (1988) also notes that because most less developed countries are agrarian societies, where agriculture is considered to be the main source of livelihood, most rural communities across Africa find the need to increase agricultural productivity through cooperatives. In Zimbabwe, rural cooperatives were promoted by the GoZ and the NGOs in the early 1980s. The scope of such initiation of rural cooperatives back then was to help people to cope with the economic, social and environmental problems. Dubell (1985) further asserts that with the establishment of rural cooperatives, most governments in Africa were able to introduce new farming and marketing techniques to the rural farmers. In this regard, rural farmers were offered more efficient agricultural and cooperative extension services, more easily and at low costs. This reflects that the group approach of cooperatives became important to extension agencies, as it proved to be easier and less expensive for such agencies to deal with groups rather than with individuals.

However, Chitsike (1988) viewed rural cooperatives as a traditional method of passing and discussing agricultural information in most agrarian societies. Such information helped farmers in their agricultural activities. As observed by Barton (2000), most of the farmers formed cooperatives with the objective of generating greater profits by obtaining inputs and services at lower costs than they could obtain elsewhere. The farmers also formed cooperatives in order to market their products at better prices and in new markets that were previously not accessible. It is also argued that cooperatives, especially in rural communities, assisted rural farmers in securing up-to-date machinery and farming technology. This has been noted to be one of the major elements that have been influencing most peasantry to join rural cooperatives in Africa. Such rural cooperatives can bring about the economies of scale, whereby people can benefit as a result of coming together and operating at a larger scale. This shows that the bigger the scale of operations of rural cooperatives become, the more the benefits the members derive. According to Schwettmann (2004), rural cooperatives are successful in economic development because they are viewed as commercial organisations that operate by a broader set of values, than those associated with the narrow pursuit of profit alone. Thus, cooperatives have been taken as business enterprises, whilst they practise economic fairness by ensuring equal access to markets and services among open and voluntary membership base.

## Research methodology and design

### Design

The research design which was used in this study is the descriptive survey. This design is considered to be the most frequently used method in educational research as it describes what the researcher sees over and beyond the horizon. Chiromo (2006) notes that a research design is concerned with turning a research question into a testing project, of which this has been considered a blue print for almost all the studies dealing with at least four problems, namely what questions to study, what data are relevant, what data to collect and how to analyse the results.

By making use of the descriptive survey, the researcher employed a mixed method approach through adopting both quantitative and qualitative approaches. Borg and Gall (1996) affirm the use of hybrid research approach in educational research by stating that many educational phenomena are best studied through a combination of the quantitative and qualitative methods. The quantitative method involved data collection procedures that resulted primarily in numerical data which was analysed by statistical methods. Dornyei (2007) took survey researches which use questionnaires as examples of the quantitative method. On the contrary, the qualitative approach in the study involved data collection procedures that resulted primarily in open and non-numerical data, which were analysed by non-statistical methods.

### Setting

**FIGURE 1:**
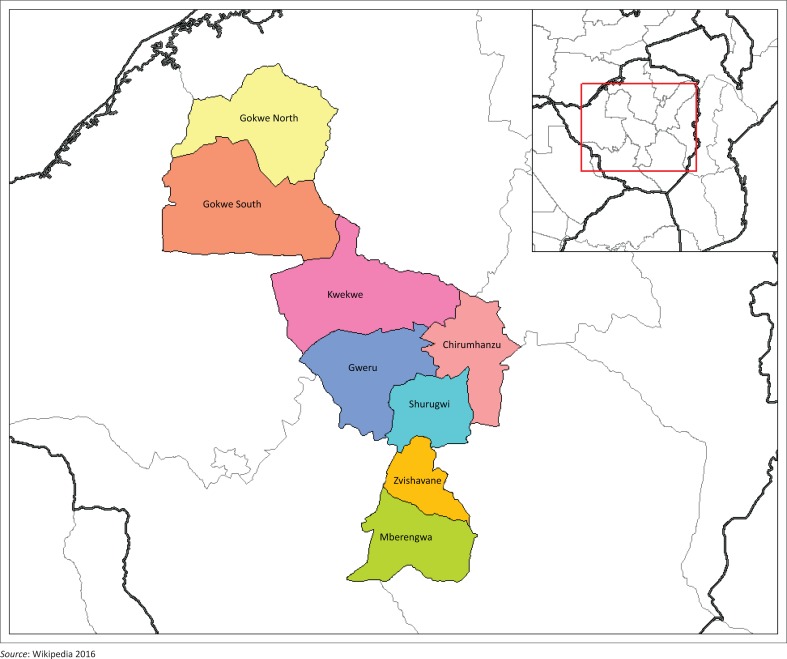
Map of Shurugwi District in the Midlands Province of Zimbabwe.

The study site for this research was Shurugwi District in the Midlands Province of Zimbabwe. The district consists of two government administrations, namely Shurugwi Town Council, which is the local authority running the affairs of Shurugwi town, and Tongogara Rural District Council, which is the local authority running the affairs of Shurugwi rural in the district. The district has an estimated total population of 99 475 people (Zimstat 2012). The central focus of the study was on the role played by rural cooperatives as a livelihood strategy adopted by the rural communities to sustain their livelihoods in order to earn a decent living. Thus, the analysis of the study focused on the activities undertaken by different cooperatives in Shurugwi District, with the aim of sustaining the livelihoods of rural communities. The district has a total of 30 cooperatives. Ten cooperatives have a focus on savings and credit, seven cooperatives specialise in fishing, six cooperatives are into livestock and crop farming and another six cooperatives are into housing, whilst one cooperative specialises in dairy farming. Figure 1 shows the map of the study site in Zimbabwe, indicating the location of Shurugwi District.

### Sampling

A sample of 50 research participants was used in the study, through purposive sampling, which is a non-probability technique. Dornyei (2007) states that in non-probability sampling technique, there are qualifying and disqualifying criteria which are used. For example, in this study the sample comprised members of the rural cooperatives in Shurugwi District, as well as government officials drawn from central and local government departments in the district. Thus, through the use of the purposive sampling procedure, the researcher managed to select a sample that was representative of the larger population. What also compelled the researcher to make use of the purposive sampling technique was that the technique gives room for one to judge whether the sample is representative of the total population based on the information supplied. In this study, the researcher selected the sample based on the information obtained from the Ministry of Small and Medium Enterprises and Cooperative Development (SMECD), a Ministry that is responsible for the registration and promotion of cooperatives in Zimbabwe. This however shows that judgment in the selection of the sample was made on the basis of the available information, and at the same time, it relied heavily on the subjectivity considerations of the researcher. Borg and Gall (1989) state that the advantage of drawing a sample from the population is that it saves the researcher a great deal of time. Working with a sample was more cost-effective than studying the entire population. It is in light of this that the sample was derived from the six rural cooperatives, namely Gutsaruzhinji Cooperative society, Makwikwi Cooperative, Tamuka Fishing Cooperative, Sunrise Savings and Credit Cooperative, Bongwe Savings and Credit Cooperative, and Shurugwi Dairy Cooperative. All these cooperatives fall under the category of primary cooperatives, as they all consist of more than five members. The cooperatives’ main purposes are to provide services and to offer employment for their members, as well as promote community development in the district. The 20% proportion in selecting the sample from each group was applied in this study.

### Procedure

The researcher made use of various sources in gathering data. As for the primary data sources, the researcher used questionnaires with open- and closed-ended questions, semi-structured interviews and observation. The questionnaire consisted of a set of questions that were put forward to participants drawn from the population under study. A total of eight government officials responded to the questionnaires which were administered for self-completion. These officials included Agricultural Extension Officers, officials from the Ministry of Small and medium enterprises and officials from the local government. The advantage of the questionnaires is that they allowed the participants to complete them at their own time. They neither had the pressure to respond to the questions quickly nor the disturbance in their daily activities. The interviews used in the study were both face-to-face and telephone interviews. Borg and Gall (1990) define an interview as a conversation which is strictly meant for the collection of information. The interviews involved direct verbal interaction between the researcher and the respondents. They were administered to 42 participants, and all these being members of the cooperatives. Among these 42 members were the six management committee members of the six cooperatives who acted as key informants. Through the use of the semi-structured interviews, the researcher was able to gather information regarding individuals’ experiences, knowledge and opinions, because the focus was on the unique meaning carried by each respondent. As such, the interviews were a very sensitive technique in uncovering some hidden issues pertaining to the role played by cooperatives among rural communities. The observation method was employed to study the daily activities of the cooperatives in order to understand their scope and focus.

## Ethical considerations

Ethics refers to the question of right or wrong, and as such ethical concerns in any research are very important as they are meant to protect the rights of participants. According to Miller (2000), ethics can be taken to refer to issues to do with morality, culture and customs, whereby the researcher in the field has to make some considerations on issues that can affect respondents. Fraenkel and Wallen (2003) indicate that it is the fundamental responsibility of researchers to do all in their power to ensure that participants are protected from physical and psychological harm, and from discomfort that may arise due to the research. Hence, it was necessary that research ethics were considered in this study. Issues of confidentiality and anonymity of the information obtained from the respondents were some aspects of ethical considerations that the researcher put into consideration. Entry points were also observed as the researcher sought permission to collect data from the Provincial Offices for the Ministry of Small and Medium Enterprises and Cooperative Development. Permission and informed consent of the participants were also sought in order for them to participate in the study.

## Results and discussion

This part focuses on the presentation of the results and their discussion. This part seeks to answer the objectives of the study, which are to identify the reasons for the establishment of rural cooperatives in the district, to assess the role played by rural cooperatives in addressing socio-economic challenges faced by rural households and to analyse the challenges faced by rural cooperatives in their attempt to address the socio-economic challenges faced by rural households.

### Reasons for the establishment of rural cooperatives in Shurugwi District

The cooperative movement in Shurugwi District started in the 1980s, with only two cooperative movements – Gutsaruzhinji and Makwikwi cooperatives. Data obtained from the field showed that the total number of cooperatives in the district now stands to 30 cooperatives. These 30 cooperatives have been sustaining the livelihoods of the rural communities through providing employment to their members. In addition, the cooperatives have provided cereal crop, dairy products, poultry products and fish products to serve the public. According to the research participants, the six major cooperatives studied in the district were established in different years. Gutsaruzhinji Cooperative was established in the year 1983, Makwikwi Cooperative in 1983, Sunrise Savings and Credit Cooperative in 2011, Bongwe Savings and Credit Cooperative in 2012, Tamuka Fishing Cooperative in 2009 and Shurugwi Dairy Cooperative in 1999 (Table 1).

**TABLE 1:** Main cooperatives in Shurugwi District and their years of establishment.

Name of cooperative	Date established	Years of operation
Gutsaruzhinji cooperative	1983	33
Sunrise Savings and Credit Cooperative	2011	5
Makwikwi cooperative	1983	33
Shurugwi dairy cooperative	2001	15
Tamuka Fishing Cooperative	2009	7
Bongwe savings and credit cooperative	2012	4

As can be observed, Gutsaruzhinji and Makwikwi cooperatives are the oldest cooperatives having been established in 1983, followed by Shurugwi Dairy Cooperative in 2001. Tamuka Fishing Cooperative which was formed in 2009, Sunrise Savings and Credit Cooperatives formed in 2011 and Bongwe Savings and Credit Cooperative created in 2012 (Table 1) were established in later years. This information implies that cooperatives play a major role in sustaining the livelihoods of rural communities and hence their continued existence. Gutsaruzhinji Cooperative, Makwikwi Cooperative and Shurugwi Dairy Cooperative have their focus on the agricultural sector. Gutsaruzhinji Cooperative offers employment, cereal crop and fresh vegetables to its members, whilst Makwikwi Cooperative also supplies cereal crop, fresh vegetables and breeds cattle, which they later sell to improve income of cooperative members. Data collected also showed that Shurugwi Dairy Cooperative provides milk to its members and also sells some of the milk to locals in order to increase the income of its members. Tamuka Fishing Cooperative provides fresh and dried fish to the cooperative members. The cooperative also sells some of the fish as a way to get income for its members. Sunrise Savings and Credit Cooperative, and Bongwe Savings and Credit Cooperative both run a scheme of lending money to their members and the public. The money that the cooperatives lend to their members and the public is paid back with interest. Apart from money lending, these two cooperatives also run poultry and goat fattening projects from which they sell chickens, eggs and goats in order to sustain the lives of cooperative members through income generated. As such, the six cooperatives studied have been able to take care of the welfare of their members. The findings are similar to those by Kopoka (2006), who noted that 75% of rural communities depended on the agricultural sector for their livelihoods. The following are the major reasons for the establishment of the above cooperatives in the district.

Data gathered from the interviews conducted with different members of the management committees of the cooperatives indicate that there were several reasons or motives as to why rural communities in Shurugwi District decided to establish cooperatives. The following are the reasons why cooperatives in the district were established (Table 2).

**TABLE 2:** Reasons for establishing cooperatives.

Reason	Frequency	Percent
Economic development – to meet members’ economic needs	12	24
Empowerment tool	09	18
To address market failures– to increase bargaining power on the market	09	18
As a defence against adverse social-economic conditions	08	16
To access cheap transport and storage facilities	06	12
To purchase bulk inputs at lower prices	06	12

*N* = 50.

From Table 2, it is clear that 24% of the respondents indicated that cooperatives in Shurugwi District were established for economic development. Other reasons for their establishment were to empower marginalised members (18%), to address market failures (18%), as a defence against adverse socio-economic conditions (16%), to access cheap transport and storage facilities (12%) and to purchase bulk inputs at lower prices (12%). It seems to achieve economic development was the main reason why the cooperatives were established in Shurugwi District. These findings concur with previous studies that cooperatives have contributed to building sustainable livelihoods by providing needed services, providing access to basic services in the community and enabling members to access and benefit from markets, and that this has resulted in members’ productive agriculture, small and medium enterprises and stable community development (Ferguson 2012).

### Roles of rural cooperatives in improving livelihoods of rural communities

This part focuses on the roles of rural cooperatives as a livelihood strategy in rural communities. In discussing these roles, specific focus is made on how rural cooperatives have helped in improving the standards of living among the rural populace. From the interviews carried out, respondents indicated the roles of cooperatives as that of employment creation, poverty alleviation, food security, women empowerment, human capital development, creation of rural markets and social integration (Table 3).

**TABLE 3:** Roles of cooperatives in improving livelihoods.

Role	Frequency	Percent
Employment creation	10	20
Poverty reduction	12	24
Improved food security	09	18
Women empowerment	07	14
Human capital development	03	06
Creation of rural market	03	06
Social integration	06	12

*N* = 50.

The major role of the cooperatives in the rural communities in Shurugwi District as indicated by the respondents is that of poverty reduction (24%), followed by employment creation (20%), improved food security (18%), women empowerment (14%), social integration (12%), creation of rural markets (6%) and human capital development (6%), as indicated above (Table 3). It was also observed by the researcher that whilst cooperatives were found to directly benefit their members, they also offered positive externalities to other members of the society. The findings that cooperatives lead to poverty reduction contradict a study by Churk (2015), who found that cooperatives played a minimal role towards promoting rural livelihoods, the feature that made poverty situation to persist to the community members. The findings by this study, that cooperatives improve the food security situation, agree with the results from a previous study, which concluded that farmer members have immensely benefitted in increasing efficiency of various agricultural inputs and overall crop productivity, making better profit through the efforts of cooperatives (Kumar et al. 2015). The next part looks at the challenges that are commonly faced by cooperatives in the rural communities of Shurugwi in their bid to sustain the livelihoods of the rural populace.

### Challenges faced by cooperatives in sustaining livelihoods of rural communities

Rural cooperatives in Shurugwi District face certain challenges in their day-to-day running of their businesses. The notable challenges that were identified by the researcher included those of poor management, small value of shares, lack of access to credit facilities and lack of access to competitive markets (Table 4).

**TABLE 4:** Challenges for cooperatives in sustaining livelihoods.

Challenge	Frequency	Percent
Poor management	10	20
Small value of shares	06	12
Lack of access to credit facilities	14	28
Lack of access to competitive markets	20	40

*N* = 50.

As can be observed (Table 4), cooperatives are faced with some challenges in sustaining the livelihoods of rural communities in Shurugwi District. As indicated by the respondents, the major challenge is the lack of access to competitive markets (40%), followed by lack of access to credit facilities (28%), poor management (20%) and the small value of shares (12%). According to the respondents, the main reason not to get competitive markets is because of low volume of production, resulting in the cooperatives only affording to serve the small local markets. Poor management implies that training programmes, which were observed to be in place in most of the rural cooperatives, are insufficient to cultivate the necessary leadership skills for proper management of the business of the cooperatives at a professional level. These findings support the previous results that for cooperatives to be viable, there is need for capable management and governance, as well as the ability to adapt to the prevailing business conditions, whereby rural cooperatives are expected to develop professional management which is democratic, inclusive, fair, transparent and with strong leadership (Mbeiyererwa 2000; Mohammed & Lee 2015). The small value of shares made it difficult for the cooperatives to have higher returns on their investments. The researcher observed that each member was entitled to contribute $10 towards the cooperative fund. The unavailability of reserve funds for most of the rural cooperatives is also one of the challenges most rural cooperatives in Shurugwi faced. According to the respondents, most cooperatives deposit their funds in banks. They further indicated that they were affected by this scenario in the sense that the real value of their money is reduced, owing to the high bank charges. The same banks according to the respondents were also reluctant to offer credit lines to the cooperatives. The above results concur with the study by Mohammed and Lee (2015), who found that cooperatives in rural areas may not prosper because of lack of working capital.

### Study trustworthiness, validity and reliability

To ensure the trustworthiness, validity and reliability of the study and data collected, the research took into consideration issues of dependability, credibility, transferability confirmability and piloting of the research instruments. This was done to fulfil the procedures of the qualitative and quantitative research. Qualitative researchers consider dependability, credibility, transferability and confirmability as trustworthiness criteria to ensure the rigour of their qualitative findings (Guba 1981; Schwandt, Lincoln & Guba 2007). Dependability was achieved through interviewing the actual members of the cooperatives in Shurugwi District. On the contrary, credibility was achieved through the process of member checking, in which the collected data was shared with the participants so that any anomalies can be rectified. As for the transferability of the study, a detailed description of the study area is tabled with sufficient information for the reader to deduce the applicability of the findings to other similar settings. Confirmability was enhanced through keeping all the data collected in a safe and retrievable place. Notes recorded from the interviews were kept in the field diary. To fulfil the procedures for quantitative research, the researcher considered the issues of study validity and reliability of the research instruments. To make the study valid, the researcher dealt with people with knowledge and experiences of running cooperatives. The research instruments, that is, questionnaires and interview guide, were tested on a small number of cooperatives members before the actual study was conducted. Any irregularities identified in the instruments were corrected.

### Practical implications

It was the researchers’ hope that upon the completion of the study, a number of players in the realm of the cooperative movement and development work in Zimbabwe and beyond would immensely benefit from the findings of the study. Policy-makers on cooperatives and those involved in rural development are expected to utilise the outcomes and recommendations to evaluate their contributions to rural development. The cooperatives members are also expected to use the findings in dealing with poverty issues in order to sustain rural livelihoods. The outcome of the research is also expected to contribute to available literature by adding new findings to the already existing body of knowledge. As such, future researchers in the field of development studies and related fields are largely expected to benefit from this study.

## Limitations to the study

In carrying out the study, the researcher faced a number of challenges. Financial resources to cover the cost of transport and to purchase research materials were one major limitation. The study was carried out over a period of 3 months. However, this period was considered to be too short for more in-depth information to be collected and analysed. The time issue was further compounded by the fact that the researcher is employed full time. This made it difficult to balance the demands of the work and the research. Despite these limitations, the researcher managed to seek financial assistance from friends and relatives for the study to be successful. On the issue of time, the researcher would in some instances make prior arrangements so that he visited the cooperatives during weekends. To save on the costs of travelling, some interviews were conducted over the phone during working hours at the respondents’ convenient times.

### Recommendations

Taking the above findings into consideration, the researcher came up with the following recommendations. Firstly, the researcher recommends that the government and the banking sector render financial support to cooperatives in rural communities to allow them to expand and diversify their business operations. It is further recommended that constant training on leadership and management skills be provided to cooperatives’ members. There is also a need for cooperatives, especially those in the agricultural sector, to form some producer associations so as to easily market their produce. Lastly, the study recommends that future research should focus on investigating issues that hinder the growth of the cooperative movement in rural communities of Zimbabwe.

## Conclusion

This study concluded that cooperatives are not a new phenomenon to exist in rural communities, as they have been part of human life in many rural areas for many previous years. Because of the cooperatives’ long existence in rural communities, the study also concluded that cooperatives play a significant role in defining and sustaining the lives of the communities. Therefore, rural communities have a lot to benefit from undertaking cooperative programmes. Rural communities that continue to exist without cooperatives are likely to find themselves entangled in high levels of poverty. The study further concluded that cooperatives are established for various purposes, including economic development, empowering of marginalised members of communities, to address market failures, as a defence against adverse socio-economic conditions, to access cheap transport and storage facilities and to purchase inputs at affordable prices. The major roles of the cooperatives to the rural communities include poverty reduction, employment creation, improved food security, women empowerment and human capital development. It was also the study’s conclusion that the running of cooperatives’ programmes is a cumbersome activity. This is so because apart from the significant role played by rural cooperatives in sustaining the livelihoods of the rural communities in Shurugwi District, most of these cooperatives face a number of challenges which prohibit them from operating at full capacity. Such challenges need to be effectively dealt with so that there is smooth running of cooperatives in rural communities. Lastly, the study concluded that cooperatives would continue to be part of rural life, as such cooperatives have a propensity to sustain livelihoods of many rural communities.
